# Practical Implementation of the Indirect Control to the Direct 3 × 5 Matrix Converter Using DSP and Low-Cost FPGA

**DOI:** 10.3390/s23073581

**Published:** 2023-03-29

**Authors:** Michal Praženica, Patrik Resutík, Slavomír Kaščák

**Affiliations:** Department of Mechatronics and Electronics, Faculty of Electrical Engineering and Information Technology, University of Zilina, 010 01 Zilina, Slovakia

**Keywords:** matrix converter, indirect control, DSP, FPGA, Simulink

## Abstract

The popularity of multiphase drives is increasing due to the growing interest in drives with more than three phases. One promising topology is the multiphase matrix converters, which enable the implementation of a single-stage AC/AC power conversion system with bidirectional power flow capability. In this paper, we present the implementation of indirect control for a practical sample of the direct matrix converter. To reduce the overall cost of the control solution for these types of converters, we utilized low-cost FPGA and DSP. The usage of only DSP itself was not possible due to low number of available PWM output needed for 3 × 5 MxC driving. Another reason is commutation, which must be precise and fast to avoid any hazardous states in the converter. Due to these problems, the authors decided to implement an algorithm of a combination of DSP and FPGA, where FPGA is used for time critical operations. The indirect algorithm treats the converter as two separate parts, the rectifier and the inverter, with the DC-LINK being fictitious. The matrix converter is composed of compact modules, and the entire system is verified. The practical verification demonstrates that matrix converters can produce a wide range of output frequencies and achieve input power factor control. Finally, we compare and review the practical model with the simulation model, examining efficiency and other parameters.

## 1. Introduction

The multiphase drives are gaining significant popularity in recent times, due to the higher torque density [1] and the property of the fault-tolerant control possibility [2,3,4,5]. This gives them a significant advantage in safety-critical applications, such as trains, elevators, cranes, and hospital applications. For supplying the multiphase machines, multiphase power electronics converters are required [6]. The cyclo-converter is a well-known type of semiconductor converter used for these types of applications [7,8,9]. The main disadvantage of the cyclo-converter is that the output frequency it produces is lower than the input frequency, and thus this type of converter is suitable only for low-frequency, low-RPM applications. Another solution is using three-phase rectifiers to rectify the input supply voltage flowing to the storage component to store energy, and finally, the n-phase output inverter, which is used to create an n-phase grid for driving the multiphase motor [10,11]. Generally, this solution is used nowadays due to the availability of the modules for these types of converters, which significantly simplifies the power electronics design. The main drawback of this solution is the presence of the storage component, in this case, the DC-Link capacitor [12,13]. It is well-known that in a harsh environment, these types of converters require much more attention and maintenance due to the failures of the capacitors. Especially, if the converter is mounted in applications where the temperature is fluctuating, the lifetime is short, or the high-cost components and advanced design solutions are required to design the converter to be able to withstand these conditions. Due to that fact, the matrix converters, which do not require any storage components in the power part, are suitable for these types of applications, where voltage source inverters may not be a good choice. As mentioned, the matrix converter utilizes the power conversion from the input to the output without using any storage components [14]. The configuration of the converter varies and can be generally referred to as m × n topology, where m is the number of input phases and n represents the number of output phases [15,16]. Another advantage of the matrix converter is its ability to change the output frequency independently of the input frequency, and thus the output frequency can be lower or higher than the input frequency of the power supply. The matrix converters have been known since the year 1980; thus, multiple control algorithms exist. The first and most well-known control algorithm developed by Venturini [17] uses the envelopes of the input voltages to switch the output waveforms. Due to this type of control, the voltage transfer of the converter is limited to the value of 0.5 [17]. In the following research, Venturini modified the control algorithm with a third harmonics injection, and the voltage transfer of this type of control for a 3 × 3 matrix converter is 0.866 [18]. Another modulation technique is the indirect control, where the matrix converter is virtually split into the rectifier stage and the inverter stage [19], which are independently controlled. In the final step, the switching signals are converted to the signals suitable for the direct matrix converter topology. Some types of matrix converters are available for purchase, and the main producer of these types of converters is the company YASKAWA, which is localized in the United States. They offer low- and high-voltage matrix converters suitable for mid- and high-power applications. The most common available configuration is 3 × 3, involving a three-phase system in the input and a three-phase system in the output of the converter. The multiphase matrix converters require more semiconductor components compared to traditional voltage source inverter (VSI), and the control algorithm increases in complexity as well. Nevertheless, despite the complexity and high semiconductor count, the matrix converters draw the attention of universities and research centers to develop matrix converters suitable for multiphase applications [20,21,22,23,24,25,26].

This paper presents the practical implementation of the indirect control of the direct topology of the matrix converter. For the power part, the compact modules presented in [27] are used to build the matrix converter in the 3 × 5 configuration. For control purposes, the control algorithm is developed on two systems, the DSP from Texas Instruments, which measures voltages and currents, calculates the duty cycles, and recognizes sectors, and the second system interconnected with DSP, based on the low-cost, low-power FPGA from Lattice Semiconductors, which applies vectors and performs fast calculations, such as matrix multiplying and executing the commutation algorithm, which is very important in the matrix converter. This system configuration was selected based on multiple reasons. First, the 3 × 5 matrix converter requires 30 PWM signals to fully control all bidirectional switches. None of the existing DSPs have that many PWM outputs available [28,29]. Although, if that many PWMs were available in the DSP, the implementation of the commutation algorithm must be implemented in software, because of the missing hardware in the PWM modulator needed for the signal swapping. Thus, software implementation would utilize too much computing power of the DSP. The authors decided to solve these problems with the combination of DSP and FPGA, where DSP calculates the mathematical equations in the control algorithm and the FPGA is used to execute the time-sensitive parts of the algorithms. The FPGA algorithm was developed using the MATLAB Simulink HDL coder, which enables fast code implementation.

The developed algorithm is evaluated in the experimental setup. First, the commutation algorithm was verified, together with indirect control itself. Finally, all output and input quantities were measured and compared to the simulation model of the matrix converter, which showed a very good match, confirming the correctness of the implemented algorithm.

## 2. Construction of the 3 × 5 Matrix Converter

The matrix converter in the 3 × 5 configuration consisted of 15 bidirectional switches in total. For every bidirectional switch, the 2 SiC transistors were used, which led to 30 transistors in the power stage in total. As mentioned in the Introduction Section, the compact modules were used to build the power stage of the converter. Every module requires a 6 PWM signal to control every transistor in the module. The signals were generated by the FPGA, which was run at the frequency of 25 MHz. Another part of the board is the DSP, the core of which was run at 90 MHz. This DSP calculated a control algorithm and sent all necessary signals to the FPGA.

The modules were interconnected by the power board, where the overvoltage protection was present. This protects the power transistors in case of failure or overvoltage generation. The protections consisted of rectifier diodes, which were connected to the output and the input of the converter. In this rectifier, the capacitors were present for the absorption of the energy, thus clamping the overvoltage. Another very important part of the converter is the input filter. Since the matrix converter is a switching converter without a DC-Link storage component, the current drawn from the supply grid has an impulse character and can disturb other devices on the same supply network. The input filter present at the input of the power converter filters the pulse current and ensures that the current drawn from the supply grid has a sinusoidal shape. For this application, the damped LC filter was selected due to its good performance, low price, and relatively low dimensions. The filter must be properly damped because the LC filter is unstable around its cut-off frequency. For the damping, the resistor parallel to the power inductor was used. This solution reliably damps the filter and does not add another parallel capacitance to the system, as in the RC parallel damping [30].

## 3. Theory of Indirect Control for the Direct Matrix Converter

**Figure 1:**
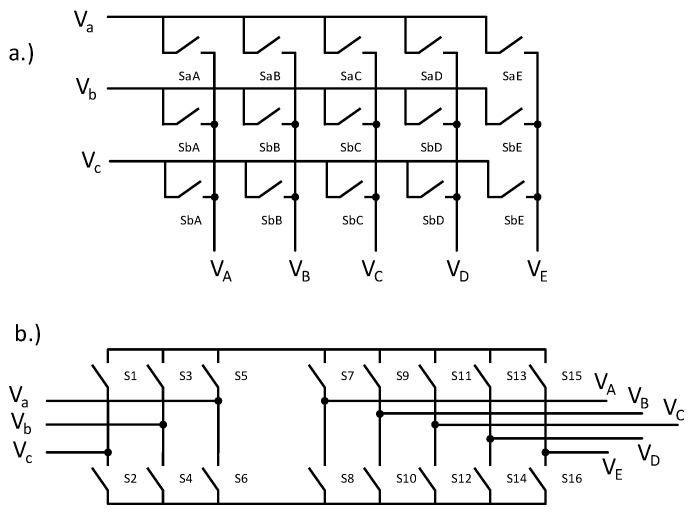
Matrix converter topology schematics: (**a**) direct and (**b**) indirect.

As was mentioned before, the indirect control will virtually split the matrix converter into two parts, a three-phase current-source rectifier at the input of the matrix converter and the five-phase voltage source inverter at the output. The schematic can be seen in Figure 1.

Since the input was a three-phase input, the hexagon can be constructed, in which the input current vector can be composed using adjacent vectors.

**Figure 2:**
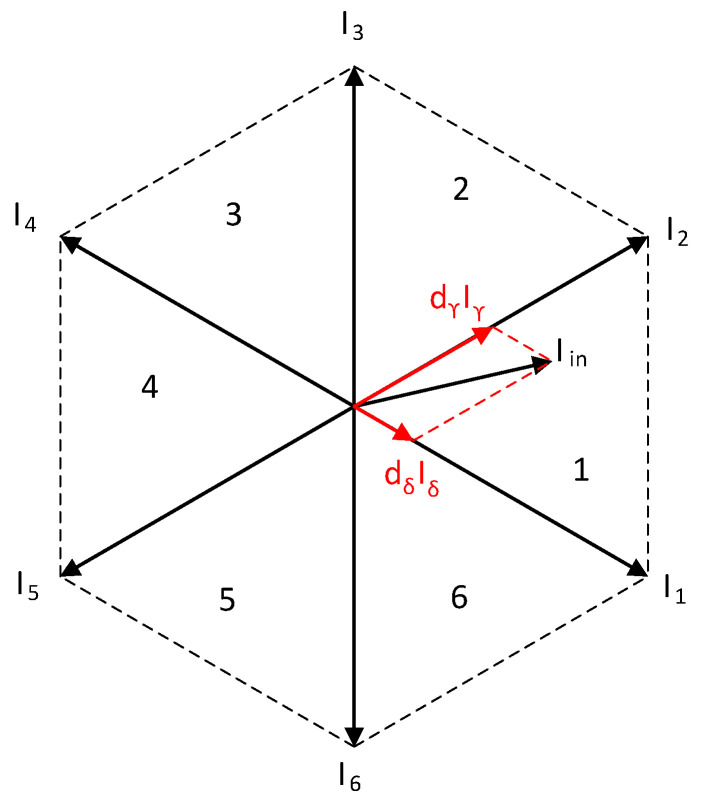
Input current hexagon.

**Figure 3:**
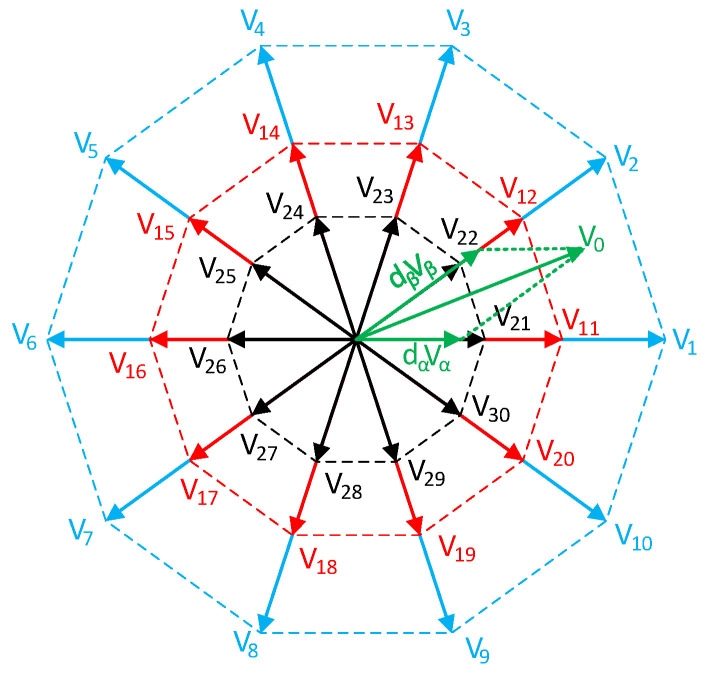
Output voltage decagon with all vectors.

From Figure 2, the equation for the input current vector, *I_in_*, applies:

(1) $$I_{in}=d_{\gamma }I_{\gamma }+d_{\delta }I_{\delta }+d_{0}I_{0}$$

where, *d_ϒ_*, *d_δ_*, and *d*_0_ represent the duty cycles of the active and zero vectors, and *I_ϒ_*, *I_δ_*, and *I*_0_ represent adjacent vectors in the sector, where the output vector, *I_in_*, lies. The duty cycles are calculated according to the following equations [31]:

(2) $$d_{\delta }=m_{r}\sin\left(\frac{\pi }{3}-\theta_{i}\;\right)$$

(3) $$d_{\gamma }=m_{r}\sin\left(\theta_{i}\;\right)$$

(4) $$d_{0}=1-d_{\gamma }-d_{\delta }$$

where *m_r_* represents the modulation index of the virtual rectifier, which can be in the range of 0–1. Similarly, for the output voltage inverter, the decagon was constructed. Since the output was five-phase output, the usable vectors were divided into large, medium, and small, according to Figure 3.

Due to the lower complexity of the control solution, only the medium and large vectors will be used in this control technique. Then, the output voltage vector can be expressed as:

(5) $$V_{O}=d_{\alpha }V_{\alpha }+d_{\beta }V_{\beta }+d_{z}V_{z}$$

where *d_α_*, *d_β_*, and *d_Z_* are duty cycles of the active and zero vectors. The duty cycles can be calculated by adjusting from three-phase to five-phase [31]:

(6) $$d_{\alpha }=m_{i}\sin\left(\frac{\pi }{5}-\theta_{i}\right)$$

(7) $$d_{\beta }=m_{i}\sin\left(\theta_{i}\right)$$

(8) $$d_{z}=1-d_{\beta }-d_{\alpha }$$

Similarly, the *m_i_* represents the modulation index of the virtual inverter. Since only medium and large vectors were used due to the simplification of the FPGA control algorithm, the duty cycles of these vectors must be calculated relative to each other, as follows:

(9) $$d_{\alpha l}=d_{\alpha }\frac{V_{l}}{V_{l}+V_{m}}$$

(10) $$d_{\alpha m}=d_{\alpha }\frac{V_{m}}{V_{l}+V_{m}}$$

(11) $$d_{\beta l}=d_{\beta }\frac{V_{l}}{V_{l}+V_{m}}$$

(12) $$d_{\beta m}=d_{\beta }\frac{V_{m}}{V_{l}+V_{m}}$$

where values of the large and medium vectors can be calculated from the distribution of the vectors in Figure 3:

(13) $$V_{l}=\frac{4}{5}\cos\left(\frac{\pi }{5}\right)V_{DC}$$

(14) $$V_{m}=\frac{2}{5}V_{DC}$$

The value of *V_DC_* is calculated from the peak input voltage, *V_in_*, the rectifier modulation index, *m_r_*, and the input displacement angle, *φ_in_*, as follows:

(15) $$V_{DC}=\frac{3}{2}V_{in}m_{r}\cos\left(\varphi_{in}\right)$$

when all the duty cycles for the rectifier and the inverter are known, vectors can be applied for known active time interval. So that this control technique can be applied for direct control, the following transformation must be calculated to transfer indirect signals to the direct signals:

(16) $$\left[\begin{array}{ccc} S_{aA} & S_{bA} & S_{cA}\\ S_{aB} & S_{bB} & S_{cB}\\ \begin{array}{c} S_{aC}\\ S_{aD}\\ S_{aE} \end{array} & \begin{array}{c} S_{bC}\\ S_{bD}\\ S_{bE} \end{array} & \begin{array}{c} S_{cC}\\ S_{cD}\\ S_{cE} \end{array} \end{array}\right]=\left[\begin{array}{cc} S_{7} & S_{8}\\ S_{9} & S_{10}\\ \begin{array}{c} S_{11}\\ S_{13}\\ S_{15} \end{array} & \begin{array}{c} S_{12}\\ S_{14}\\ S_{16} \end{array} \end{array}\right]\left[\begin{array}{ccc} S_{1} & S_{3} & S_{5}\\ S_{2} & S_{4} & S_{6} \end{array}\right]$$

## 4. Practical Implementation of the Indirect Control

**Figure 4:**
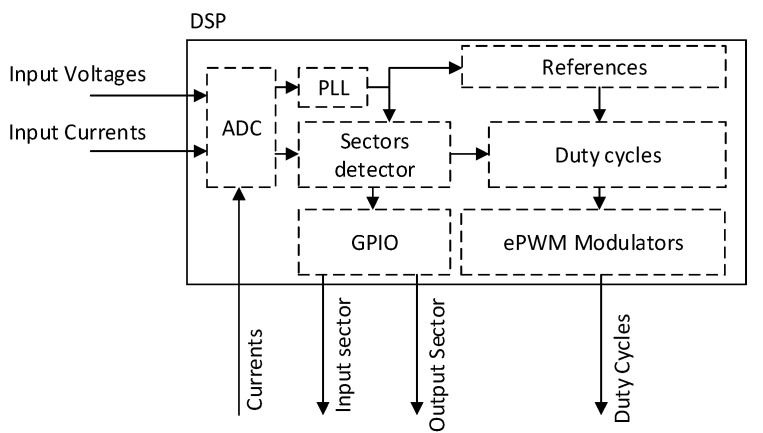
Block diagram of the DSP control algorithm.

The control algorithm was divided into two parts, according to the system that is performing the calculations. Since the DSP has A/D converters built-in, it measures the input voltages, currents, and output currents. The measured voltage at the input of the converter was used to run the PLL block, which generates the reference angle in phase with the input power supply. This block is necessary because the internal references generated by the DSP for the output waveform must be the same or multiply the frequency of the input waveform. After references were generated, the DSP detected in which sectors the input current and output voltage lie. After the sector is known, the duty cycles, according to the Equations (2)–(4) and (6)–(12), could be calculated. The block diagram of the DSP control algorithm can be seen in Figure 4.

The actual sector number for the rectifier and inverter is encoded to the GPIO pins as the binary number, which can be processed fast by the FPGA. The calculated duty cycles according to the Equations (2)–(4) were modulated using PWM modulators in the DSP to transfer the information to the FPGA that is selecting the vectors. For the PWM, the up–down type was used due to its advantage of transferring the symmetric sequence of the signal. The selected sequence for the rectifier was the following:

(17) $$\frac{d_{0}}{4}\;,\frac{d_{\delta }}{2}\;,\frac{d_{\gamma }}{2}\;,\frac{d_{0}}{2}\;,\frac{d_{\gamma }}{2}\;,\frac{d_{\delta }}{2}\;,\frac{d_{0}}{4}\;,\;\;$$

**Figure 5:**
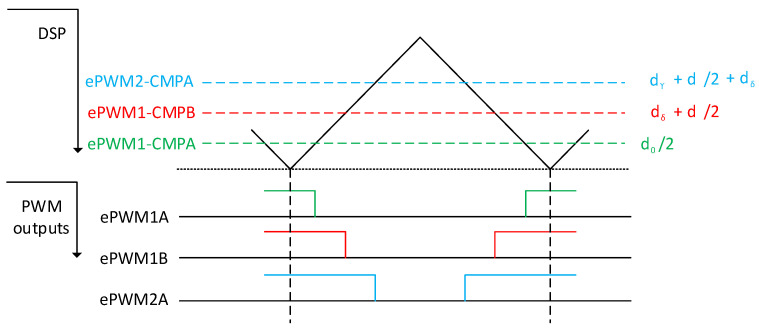
Process of the encoding vector times for the rectifier to the PWMs.

The sequence was selected to be symmetrical, which ensures better waveform quality and THD of the current. The values for the duty cycles calculated by Equations (2)–(4) were multiplied by the ePWM modulator constant and fed to the PWM modulator. The output of the DSP modulator generated the following patterns, which are shown in Figure 5.

The vectors for the rectifier part of the indirect matrix converter are summarized in Table 1.

The individual switches states for every vector are defined as in Table 2.

Similarly, for the inverter stage, the approach was the same as for the rectifier. If the output sector of the inverter was odd, the following sequence of the vectors was applied:

(18) $$\frac{d_{z1}}{2}\;,\frac{d_{\alpha m}}{2}\;,\frac{d_{\beta l}}{2}\;,\frac{d_{\alpha l}}{2}\;,\frac{d_{\beta m}}{2}\;,\frac{d_{z2}}{1}\;,\frac{d_{\beta m}}{2}\;,\frac{d_{\alpha l}}{2}\;,\frac{d_{\beta l}}{2}\;,\frac{d_{\alpha m}}{2}\;,\frac{d_{z1}}{2}\;,\;\;$$

The sequence was again chosen to be symmetrical to ensure a better output voltage THD. If the output sector was even, the following sequence applied instead of the previous one:

(19) $$\frac{d_{z1}}{2}\;,\frac{d_{\beta m}}{2}\;,\frac{d_{\alpha l}}{2}\;,\frac{d_{\beta l}}{2}\;,\frac{d_{\alpha m}}{2}\;,\frac{d_{z2}}{1}\;,\frac{d_{\alpha m}}{2}\;,\frac{d_{\beta l}}{2}\;,\frac{d_{\alpha l}}{2}\;,\frac{d_{\beta m}}{2}\;,\frac{d_{z1}}{2}\;,\;\;$$

**Figure 6:**
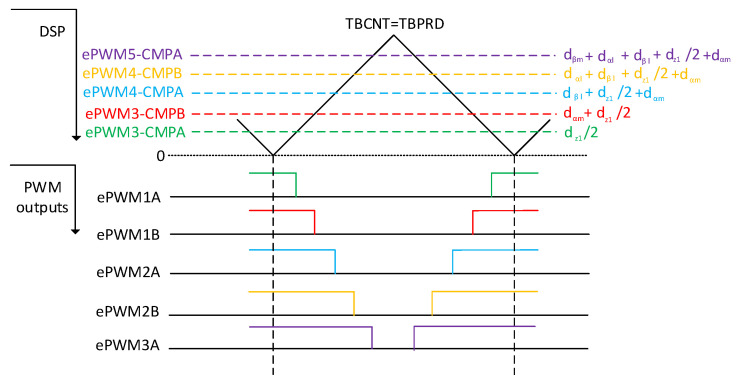
Process of the vector times encoding for the output rectifier to the PWMs.

By alternating between sequences in Equations (18) and (19) according to the evenness or oddness of the inverter sector, the number of the bidirectional switch turns was reduced, so the minimum switching control using space vectors was achieved. The PWM signals at the output of the DSP for the sequence in Equation (18) are shown in Figure 6.

The vectors for the inverter with alternating even/odd sequences are summarized in Table 3. The vectors were applied according to the sequences shown in Equations (18) and (19). The switches state according to the vectors in Table 3 is summarized in Table 4.

**Figure 7:**
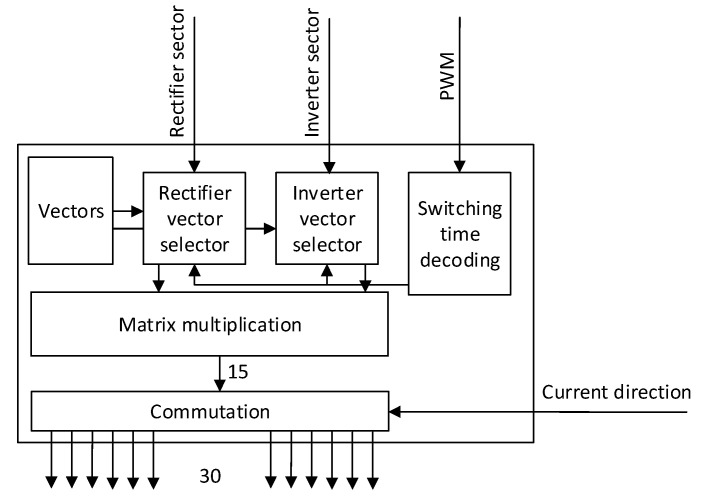
Block diagram of the control FPGA.

Table 4 summarizes the logical states of the top switches in the inverter stage according to Figure 1b. The logical state of the bottom switches: S8–S10–S12–S14–S16, is the inversion of the switches: S7–S9–S11–S13–S15, respectively, which are shown in Table 4. The PWM signals shown in Figure 5 and Figure 6 were then fed to the FPGA. In total, three signals are needed for rectifier sectors, four signals for the inverter sectors, three PWM signals for the rectifier times, and five signals for the inverter times’ definition. In addition, the synchronization signal was added for DSP and FPGA synchronization. This pulse is generated whenever the ePWM counter equals zero, to synchronize both devices at the beginning of every switching period. The switching frequency of the presented matrix converter was 10 kHz. The FPGA board was designed on a separated PCB, where all required power supplies and voltage shifters were presented. The FPGA calculated the on-time signals from the PWM signals and applied the correct vectors, defined by the sector’s signals. The block diagram of the FPGA algorithm is shown in Figure 7.

**Figure 8:**
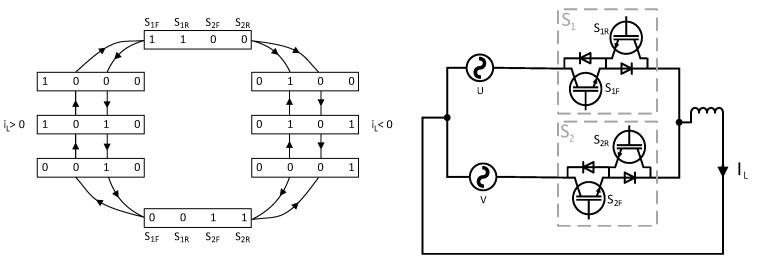
Flow diagram of the four-step commutation for positive and negative output currents.

The matrix multiplication converts the signals from the rectifier and inverter to the signals suitable for the direct matrix topology, according to Equation (16). The used commutation in this experimental application was a four-step current commutation, which determined the output switches’ status based on the direction of the output current of the module. Every module has its own built-in current direction detection circuit. The four-step commutation was selected due to its reliability and robustness against noise at the output. The FPGA was run at the frequency of 25 MHz, and one step of the commutation was set to 4 clock pulses of the oscillator. Thus, one commutation step lasted 160 ns, and the whole four-step commutation lasted 640 ns. This low time was selected due to the fact that the power devices were SiC MOSFET transistors, which have low turn-on and turn-off times. The commutation block in the FPGA generated all 30 switching signals for all the power semiconductors in the direct matrix converter. Additionally, the commutation algorithm included the current change direction during the commutation process protection. In other words, at the beginning of the commutation, the sample of the current direction was taken and stored during the commutation process. Thus, if the current changes in the middle of the commutation process, the process is not interrupted and is finished without glitches. The sequence of the commutation process in both current directions is depicted in Figure 8.

For the practical implementation of the commutation and vector handling algorithm, the low-cost iCE family from the Lattice manufacturer was used. Specifically, FPGA iCE40HX1K with the 1280 microcell was used, and the algorithm used 38% of the device capacity. This means that the device can easily handle more tasks if necessary and can be used to implement other types of control algorithms. The supply voltage of the FPGA is 1.8 V for the core and 3.3 V for GPIO pins. The device does not have an internal FLASH memory, so the external one with 32 Mbit of available capacity was used. This memory is programmed using a simple SPI programmer, so it does not require a special costly programmer. The algorithm for the FPGA was designed using the MATLAB Simulink HDL Coder environment, where a block diagram of the control and commutation was designed. Then, Verilog code was generated, and using a tool from the FPGA manufacturer, the binary file was created and used to program the onboard FLASH memory. Table 5 summarizes the FPGA usage of the algorithm as shown in Figure 7.

**Figure 9:**
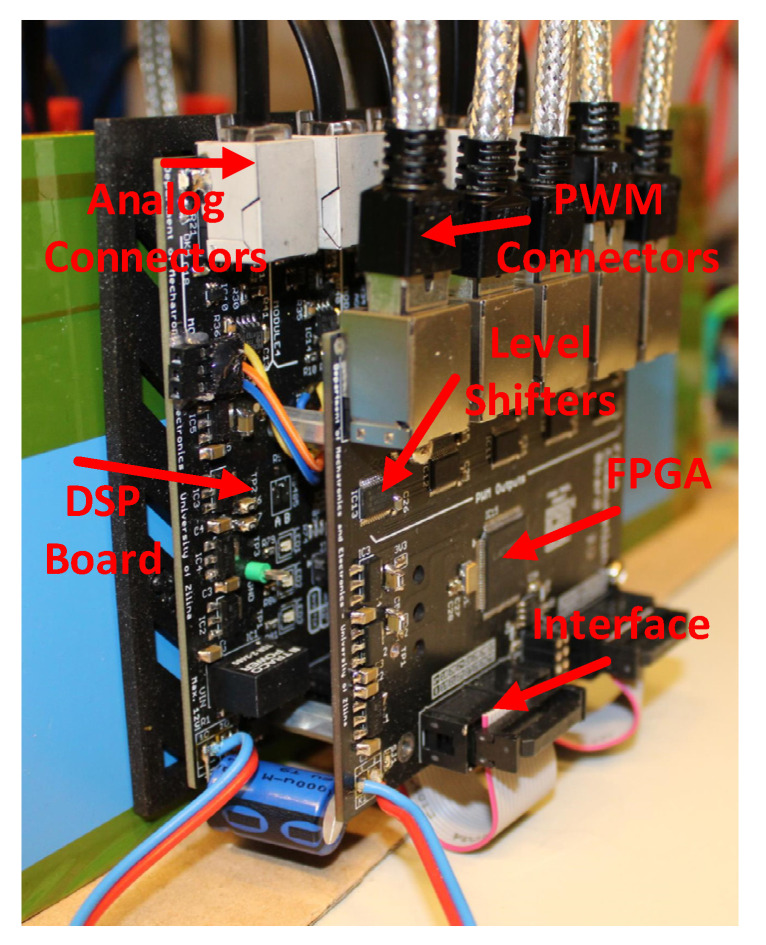
Control boards (DSP and FPGA).

The constructed control board with DSP and FPGA is shown in Figure 9.

**Figure 10:**
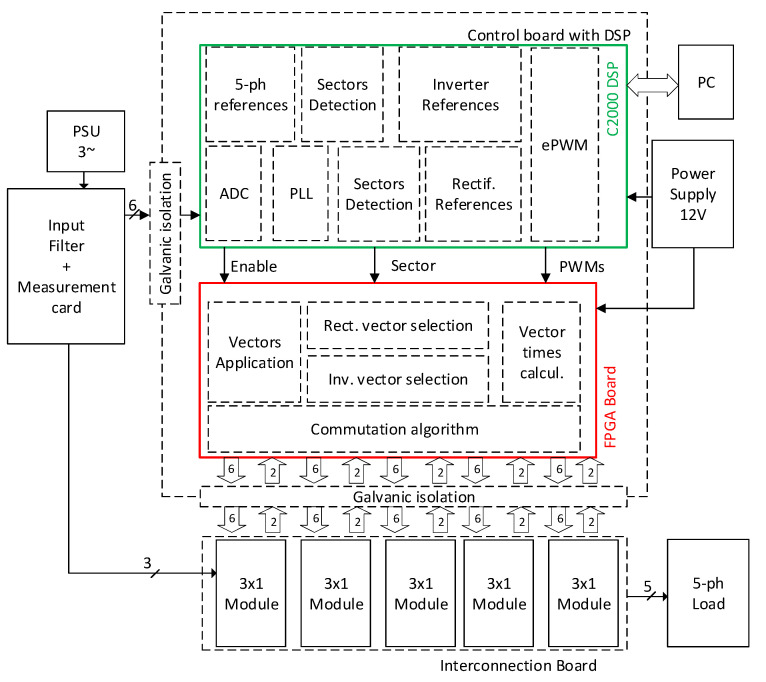
Block diagram of the presented 3 × 5 matrix converter prototype.

The block diagram of the final solution presented in this paper is shown in Figure 10.

**Figure 11:**
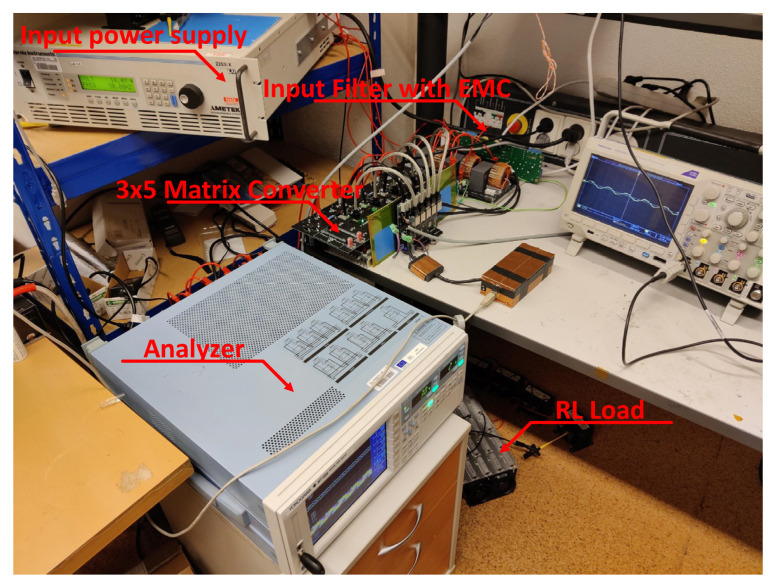
Test setup for the implemented control verification.

The circuit in Figure 10 shows a total circuit block diagram of the measurement shown in Figure 11. The PSU, input filter, all five modules, and the five-phase load share common potential, which was created by the three-phase power supply. Control cards, composed of DSP and FPGA cards, were powered by the galvanically isolated 12 V power supply, and all signals were galvanically separated from the power circuit as well. The combination of the DSP and FPGA was used due to multiple reasons. The standard MCUs have a limited number of PWM outputs, because this 3 × 5 topology requires 30 PWM signals to control all transistors. Another reason is that the order of the pulses must change with the current direction at the output of the matrix converter. This change must be detected and executed as fast as possible and cannot be implemented only in the software of the DSP. Hardware PWM modulators do not allow this change; thus, this process was implemented in the FPGA due to the hardware implementation and execution speed, which are crucial in commutation.

## 5. Practical Verification of the Indirect Control

For testing purposes, the presented modules were arranged in the 3 × 5 configuration. In total, five modules were required to build a matrix converter in the presented configuration. At the input of the matrix converter, the LC filter was connected to filter the switched current. As the power supply, the California Instruments 2253iX was used. As the load, the passive RL load with R = 7.8 Ω and L = 30 mH and active cooling were utilized. This load can handle up to 500 W of continuous power and 1 kW of pulsed power for a maximum period of 3 min. As the output analyzer, the YOKOGAWA WT1800 six-channel power analyzer was used to analyze the output waveforms and parameters of the switched voltage and current and to calculate the output power to analyze the efficiency of the matrix converter. The experimental setup can be seen in Figure 11.

**Figure 12:**
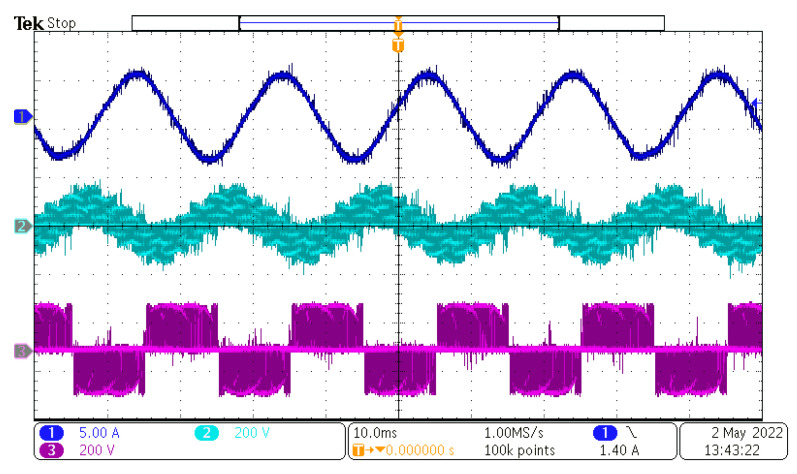
Measured results at f_IN_ = 50 Hz and f_OUT_ = 50 Hz. CH1—output current, CH2—output load voltage, CH3—output phase to phase voltage.

**Figure 13:**
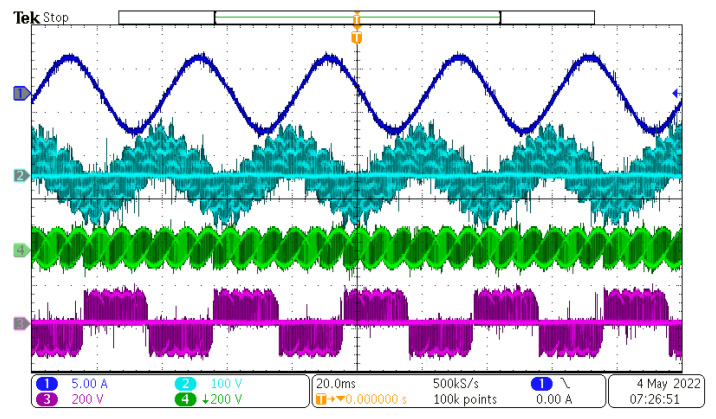
Measured results at f_IN_ = 50 Hz and f_OUT_ = 25 Hz. CH1—output current, CH2—output load voltage, CH3—output phase to phase voltage, CH4—output phase to input neutral voltage.

**Figure 14:**
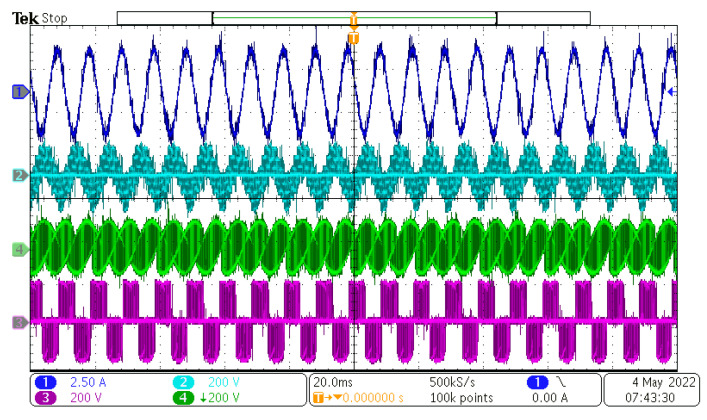
Measured results at f_IN_ = 50 Hz and f_OUT_ = 100 Hz. CH1—output current, CH2—output load voltage, CH3—output phase to phase voltage, CH4—output phase to input neutral voltage.

**Figure 15:**
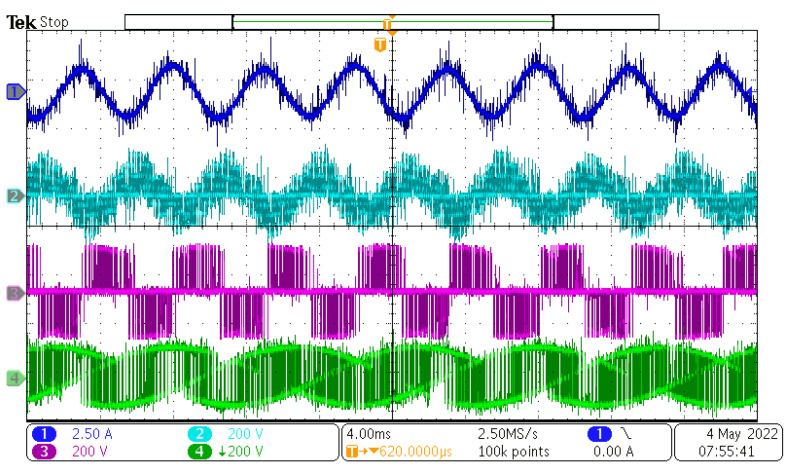
Measured results at f_IN_ = 50 Hz and f_OUT_ = 200 Hz. CH1—output current, CH2—output load voltage, CH3—output phase to phase voltage, CH4—output phase to input neutral voltage.

After the power supply output was powered on, the control DSP started the PLL sequence to detect the input phase, frequency, and amplitude. After successful detection, the output references were generated and duty cycles with the input/output vectors were calculated and determined. The value of the rectifier modulation index, m_R_, was set to 1, and the value of the inverter modulation index, m_I_, changed in range from 0.1 to 1.6. Inverter modulation index 1,6 is the maximum value, because higher values overmodulate the inverter part of the MxC. The measured results can be seen in Figure 12, Figure 13, Figure 14 and Figure 15.

**Figure 16:**
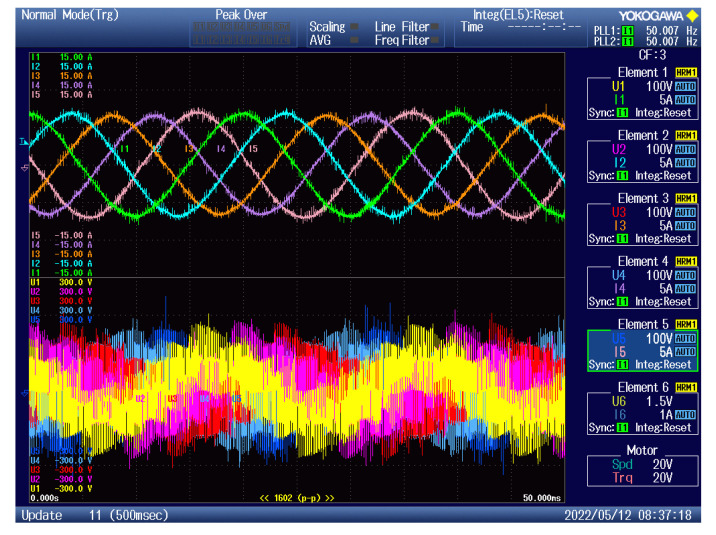
Measured results from the YOKOGAWA analyzer: top waveform—output phase current, bottom waveform—output phase voltages.

As can be seen, the output current of the converter had a sinusoidal shape, as expected. The output frequency can be changed regardless of the input frequency, as can be seen in Figure 12, Figure 13, Figure 14 and Figure 15. Additionally, all the output currents and the output voltages were measured using the YOKOGAWA analyzer. The results can be seen in Figure 16. The measured current at the output of the converter was sinusoidal, with an even phase shift between each phase, 72°. The output voltage has a switching nature, but due to the inductive nature of the load, the current was sinusoidal, with a measured THD of 5.2%. Thus, the matrix converter is suitable for applications where multiphase drives need to be powered. Additionally, the matrix converter can generate more output phases than are available at its input.

**Figure 17:**
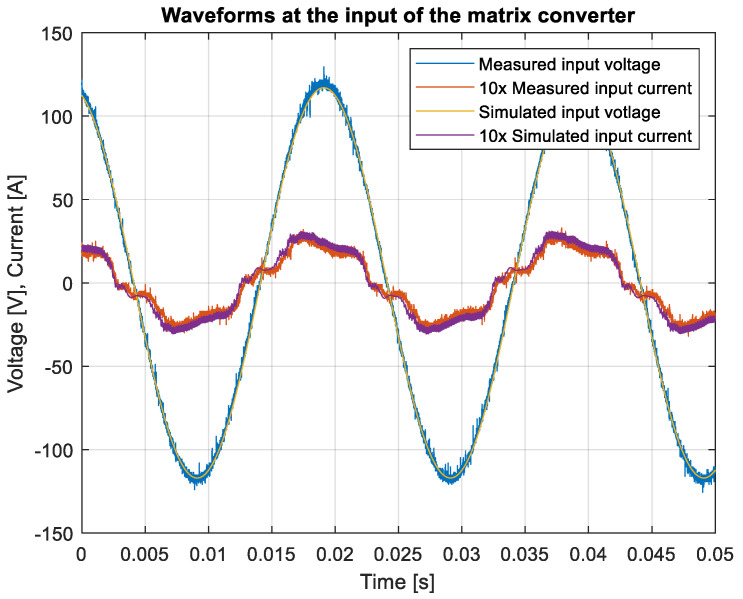
Comparison of the measured and simulated waveforms.

Due to the verification of the practical model, the simulation in the MATLAB Simulink environment was created to confirm the practical results and compare them to the simulated ones, to verify the behavior of the built sample of the matrix converter. Additionally, the model in the MATLAB Simulink environment had parameters of the used transistor implemented, so the efficiency of the simulated and measured models can be compared, too. The input waveforms of the simulation and the practical measurement are compared in Figure 17.

**Figure 18:**
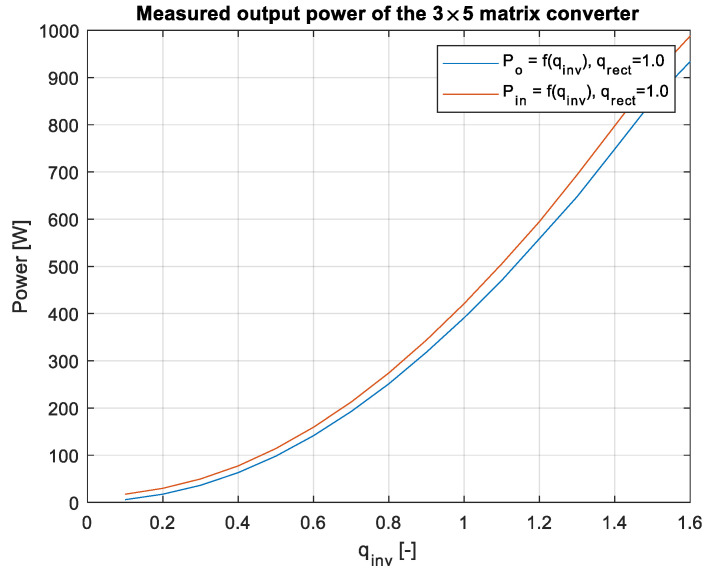
Input and output power as a function of inverter gain.

As can be seen in Figure 17, the measured and simulated waveforms were identical. The sinusoidal shape of the current is caused by the input filter, filtering the switched current. Finally, the efficiency of the converter was measured using the input power supply and the output power analyzer. The gain of the rectifier was set to one and the gain of the inverter was adjusted from 0.1 to 1.6, with the steps of 0.1. The input and the output power of the converter are shown in Figure 18.

**Figure 19:**
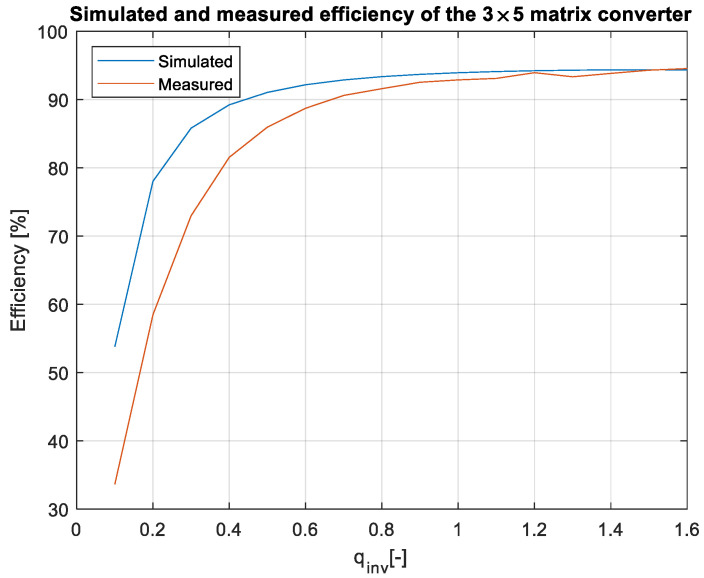
Comparison of simulated and measured data.

From the measured data in Figure 18, the efficiency characteristics of the matrix converter can be plotted. Similarly, the same conditions in which the converter was measured were simulated to compare the efficiency results, as shown in Figure 19.

**Figure 20:**
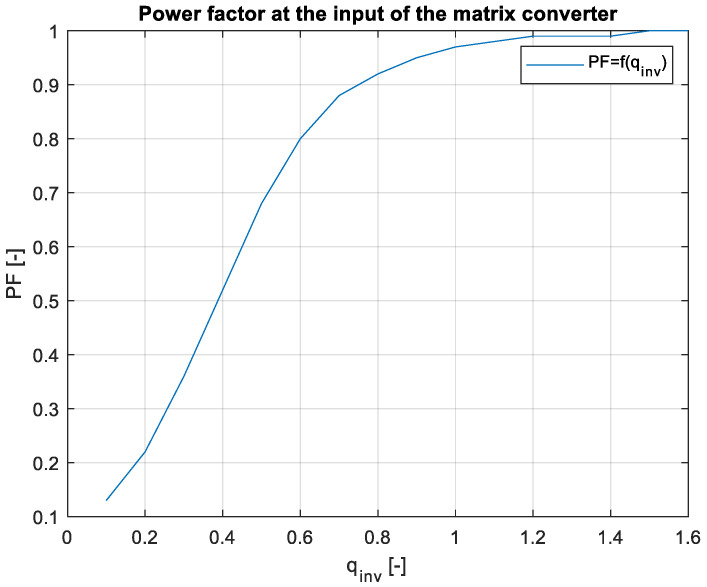
PF at the input of the MxC.

As can be seen in Figure 19, the measured and simulated efficiency were very similar. At the low inverter gain values, the difference between the model and practical sample was bigger. This is caused by the measurement, because at the low output powers, the output current waveforms were very noisy, which caused an error in the output power measurement, and thus, the measured efficiency was lower. With the increasing inverter gain, as the output power increased, the error between the model and the practical sample was lower, whereas, at the power of 930 W, the difference was 0.2%. The measured efficiency of the converter was 94.5% and the simulated efficieny was 94.3%. The output power in this measurement was from 6 W to 930 W, which relates to the values of the gain from 0.1 to 1.6, as shown in Figure 18. Finally, the most important aspect of the matrix converter, and in general in all the AC-powered converters, is the input power factor. The measured power factor of the 3 × 5 matrix converter can be seen in Figure 20.

At the low-output powers, the PF at the input was low because of the effect of the input filter. The input filter has a capacitive character to the grid, so it caused the phase shift, thus lowering the power factor value. With the rising output power, the power factor was also rising, and at approximately around 200 W, it reached a value of 0.9, and at the output powers above 450 W, the input PF was 0.96, with a maximum value of 1 at the output power of 930 W. This means the converter was drawing only the active power from the grid, without any reactive or deformation power components. If the input filter of the converter was designed to the nominal power of the converter, the input power factor could be very close to unity.

## 6. Conclusions

In this paper, the practical implementation of the indirect control for the direct matrix converter was presented and practically verified. The control algorithm was implemented using a DSP for measurement and calculations, and low-cost FPGA for time-sensitive processes, such as vectors’ implementation and commutation of the switches. For this verification, the four-step current commutation was used due to its robustness and relatively easy implementation, which requires only the current direction detection circuit, which was already implemented in the power modules. The symmetric sequence together with vector order alternating ensured minimum switching pulses, thus lowering the switching losses in the converter. The performance of the practical sample was investigated together with a comparison with the simulated model. The maximum efficiency of the physical model was 94.5%, whereas the simulated model showed a maximum efficiency of 94.3%, which is a 0.2% difference, showing very good agreement between the practical and simulation models. The maximum efficiency was achieved at the output power of 930 W. The unity power factor can be achieved due to the possibility of controlling the input power factor of the matrix converter. Figure 12, Figure 13, Figure 14 and Figure 15 showed the output quantities from the experimental model of the matrix converter. The output current was sinusoidal, with a small THD (5.2%) and with the expected shape of the voltage waveforms. Additionally, the input signals showed very good matching with the simulation modes, as shown in Figure 17. During the verification, the gain of the inverter stage was changed from 0.1 to 1.6 to set the output power. The results from the measurement were shown in Figure 18, together with efficiency results in Figure 19. The input power factor was load-dependent, as shown in Figure 20, where at the low-input powers, the PF was low, but with the rising output power, the influence of the input filters became neglectable, and the converter achieved the unity power factor.

The main advantage of the matrix converters is the absence of the DC-Link capacitor, which makes this type of converter more reliable and suitable for military, aerospace, or medical applications. In future work, the experimental matrix converter will be tested in combination with the five-phase induction machine to evaluate the behavior of the matrix converter during the dynamic loads.

## Figures and Tables

**Table 1:** The layout of the rectifier vectors according to the sector.

Rectifier Sector	I_δ_	I_ϒ_	I_0_
1	I_1_	I_2_	I_7_
2	I_2_	I_3_	I_9_
3	I_3_	I_4_	I_8_
4	I_4_	I_5_	I_7_
5	I_5_	I_6_	I_9_
6	I_6_	I_1_	I_8_

**Table 2:** Definition of the rectifier vectors.

Vector/Switch	S_1_	S_3_	S_5_	S_2_	S_4_	S_6_
1	1	0	0	0	1	0
2	1	0	0	0	0	1
3	0	1	0	0	0	1
4	0	1	0	1	0	0
5	0	0	1	1	0	0
6	0	1	1	0	0	0
7	1	0	0	1	0	0
8	0	1	0	0	1	0
9	0	0	1	0	0	1

**Table 3:** The layout of the inverter vectors according to the sector.

Sector	V_α m_	V_α l_	V_β m_	V_β l_	V_z1_	V_z2_
1	V_11_	V_1_	V_12_	V_2_	V_31_	V_32_
2	V_13_	V_3_	V_12_	V_2_		
3	V_13_	V_3_	V_14_	V_4_		
4	V_15_	V_5_	V_14_	V_4_		
5	V_15_	V_5_	V_16_	V_6_		
6	V_17_	V_7_	V_16_	V_6_		
7	V_17_	V_7_	V_18_	V_8_		
8	V_19_	V_9_	V_18_	V_8_		
9	V_19_	V_9_	V_20_	V_10_		
10	V_11_	V_1_	V_20_	V_10_		

**Table 4:** Definition of the inverter vectors.

Vector/Switch	S_7_	S_9_	S_11_	S_13_	S_15_
1	1	1	0	0	1
2	1	1	0	0	0
3	1	1	1	0	0
4	0	1	1	0	0
5	0	1	1	1	0
6	0	0	1	1	0
7	0	0	1	1	1
8	0	0	0	1	1
9	1	0	0	1	1
10	1	0	0	0	1
11	1	0	0	0	0
12	1	1	1	0	1
13	0	1	0	0	0
14	1	1	1	1	0
15	0	0	1	0	0
16	0	1	1	1	1
17	0	0	0	1	0
18	1	0	1	1	1
19	0	0	0	0	1
20	1	1	0	1	1
31	0	0	0	0	0
32	1	1	1	1	1

**Table 5:** FPGA usage summary.

Category	Available on FPGA	Used by the Algorithm
Logic Cells	1280	325
PLBs	160	81
I/Os	72	55

## Data Availability

Data is contained within the article.
